# Potential Antiviral Action of Alkaloids

**DOI:** 10.3390/molecules27030903

**Published:** 2022-01-28

**Authors:** Frage L. Abookleesh, Bader S. Al-Anzi, Aman Ullah

**Affiliations:** 1 Department of Agricultural, Food and Nutritional Science, University of Alberta, Edmonton, AB T6G 2R3, Canada; abooklee@ualberta.ca; 2 Department of Environment Technologies and Management, Kuwait University, P.O. Box 5969, Kuwait City 13060, Kuwait; bader.alanzi@ku.edu.kw

**Keywords:** antiviral, alkaloids, natural products, viral entry, viral replication, inhibition, protein synthesis, DNA, RNA

## Abstract

Viral infections and outbreaks have become a major concern and are one of the main causes of morbidity and mortality worldwide. The development of successful antiviral therapeutics and vaccines remains a daunting challenge. The discovery of novel antiviral agents is a public health emergency, and extraordinary efforts are underway globally to identify safe and effective treatments for different viral diseases. Alkaloids are natural phytochemicals known for their biological activities, many of which have been intensively studied for their broad-spectrum of antiviral activities against different DNA and RNA viruses. The purpose of this review was to summarize the evidence supporting the efficacy of the antiviral activity of plant alkaloids at half-maximum effective concentration (EC_50_) or half-maximum inhibitory concentration (IC_50_) below 10 μM and describe the molecular sites most often targeted by natural alkaloids acting against different virus families. This review highlights that considering the devastating effects of virus pandemics on humans, plants, and animals, the development of high efficiency and low-toxicity antiviral drugs targeting these viruses need to be developed. Furthermore, it summarizes the current research status of alkaloids as the source of antiviral drug development, their structural characteristics, and antiviral targets. Overall, the influence of alkaloids at the molecular level suggests a high degree of specificity which means they could serve as potent and safe antiviral agents waiting for evaluation and exploitation.

## 1. Introduction

Viral epidemics and pandemic diseases have threatened humanity historically and have had significant impacts on human society [1]. Viruses, followed by some bacteria and protozoa, have been classified as the key pathogens causing human illnesses since 1973 [2]. Viruses are known for serious infectious diseases they cause, including human immunodeficiency virus (HIV), hepatitis B and C (HBV and HCV), coronaviruses including Middle Eastern respiratory syndrome (MERS) and extreme acute respiratory syndrome (SARS), influenza (seasonal), smallpox, viral hemorrhagic fever (Ebola), dengue fever, and chikungunya virus [3,4]. New human virus species are still being detected, at a rate of three or four per year, and over two-thirds of all new human pathogens are viruses. In 2019, there were 690,000 deaths due to AIDS-related illnesses worldwide, compared with 1.7 million in 2014 [5]. A newly detected coronavirus-induced disease (COVID-19) was declared a pandemic in 2020 and has seriously affected the world population, resulting in over 216,074,000 infected cases and 4,496,998 deaths until 27 August 2021, with a notable upward trend [6].

Viruses, particularly the rapidly mutating ones, pose a great pandemic threat to human health especially due to the limited number of vaccines and antiviral drugs. However, many antiviral drugs have been developed since the 1960s and have helped the world to coexist with various viral infections [7]. Over the past 30 years, about 50% of approved medicines have been produced from natural products, either directly or indirectly [8]. This stems from a long tradition of treating various diseases using particular types of plants (herbs) as medical therapeutics.

Alkaloids are a large and structurally diverse group of natural products of microbial, plant, and animal origin. Alkaloids are usually nitrogenous small organic molecules of plant origin, and about 20% of plant species contain alkaloids [9]. Several families of the plant kingdom, such as the Ranunculaceae, Papaveraceae, Fabaceae, Loganiaceae, and Menispermaceae, contain alkaloids [10]. Among other bioactive ingredients of medicinal plants, natural alkaloids have been successfully developed into chemotherapeutic drugs such as camptothecin (CPT), a well-known topoisomerase I inhibitor and vinblastine, which is a highly successful antitumor drug. Similarly, quinidine is used for restoring normal sinus rhythm, treating atrial fibrillation and flutter, as well as ventricular arrhythmias. Another alkaloid named papaverine is used to treat many types of smooth muscle spasms, such as “vascular spasms” associated with acute myocardial infarction and angina pectoris, as well as visceral spasms [11,12]. Alkaloids also exhibit different biological activities, for instance, antitumor, antidepressant, anti-inflammatory, anti-angiogenic, and anti-dementia [13,14,15,16]. In this review, we summarize the experimental evidence for the antiviral activity of natural alkaloids with a half-maximal effective concentration (EC_50_) or half-maximal inhibitory concentration (IC_50_) below 10 μM. We also highlight the molecular sites most often targeted by natural alkaloids acting against different virus families.

## 2. Research Methodology

Pertinent literature was obtained by concomitantly searching the words “bioactive, alkaloid/phytochemicals”, “antiviral”, and “activity” in the Google scholar, Pub Med, Science Direct, Scopus, SpringerLink, Web of Science databases. The literature search was independently performed by one researcher. One hundred forty- two articles from 1950 up to 2021, were selected, cited, and carefully analyzed in order to find data related to the topic and aims of this review. All articles and information used for this review were either published or accepted for publication in English language peer-reviewed journals. Mendeley desktop software was used for preparing the bibliography. The search criteria used were: (1) the antiviral potential of natural alkaloid, (2) isolated phytochemicals alkaloids, or its derivatives effective against DNA and RNA viruses, and (3) antiviral activities of alkaloids that show at a half-maximal effective concentration (EC_50_) or half-maximal inhibitory concentration below (IC_50_) below 10 μM with no significant toxicity. However, the data from only 25 investigated pure compounds through in vitro and in vivo studies met the inclusion criteria of this review. Exclusion criteria: (1) antiviral activities with an inhibitory concentration (EC_50_, IC_50_) higher than 10μM, (2) literature duplication, (3) non-relevant articles, and (4) studies involving plant extracts with undefined active compounds.

## 3. Natural Products as a Source for Antiviral Drugs Development

Although vaccinations have been very effective in controlling many viral diseases, some infections can probably be treated only by antiviral chemotherapy. Antiviral drugs could provide the best protection strategy against a virus outbreak which is a permanent threat. Various FDA approved antiviral drugs are available for the inhibition and treatment of virus-related infections, such as rimantadine and amantadine for the inhibition and treatment of influenza A virus, and acyclovir and vidarabine against the varicella-zoster virus and herpes simplex virus infections. Similarly, ribavirin is used against the respiratory syncytial virus and valaciclovir as well as famciclovir against varicella-zoster virus infections. Notwithstanding its benefit, antiviral chemotherapy has some restrictions, namely a narrow antiviral range, ineffectiveness against a dormant virus, and evolution of drug-resistant mutants [7,17].

Natural products have played a significant role in drug development. There could be no accurate and comprehensive description of the role of natural products in drug discovery without the mention of alkaloids. The use of natural products began with the discovery and isolation of morphine from the opium poppy in 1803 [18]. There are several historical examples in which the therapeutic agent present in a natural remedy helped to reveal new physiological features; for instance, morphine enabled an understanding of the opiate receptors and the pathways of endorphin and enkephalin [19]. Recent trends in the pharmaceutical industry have become increasingly focused on small molecules from plants as a source of potential antiviral agents in the context of an urgent need for new therapeutic agents due to the issues of drug resistance and side effects of some drugs [20]. The application of advanced technologies and methods in molecular biology as well as reverse genetics has dramatically increased the possibility of conducting screening of new phytochemical compounds from plant extracts [21].

Since 1952 numerous research efforts have been made to determine the antiviral efficacy of medicinal plants [22]. Subsequent studies have investigated the extracts of several medicinal plants for their antiviral activities. For instance, an investigation into traditional medicinal plants in Togo revealed ten different plants that exhibited antiviral activities against herpes simplex, sindbis virus, and poliovirus [23]. The antiviral activities of nine Malagasy plants have been reported as having a significant impact on the herpes simplex virus (HSV), and four plants have been described as acting against the sindbis virus [24]. A total of 105 plant species have been identified and reported for their potential antiviral activities against a wide range of viruses [25]. The most recently adopted strategy for employing natural products in drug discovery involves moving from a broader approach to a narrower and more applicable one using standard procedures that involve the isolation, characterization, and identification of potent phytochemicals followed by in vitro and in vivo assays to establish the most promising phytochemicals before a clinical trial begins [26]. The commonly isolated and identified phytochemicals from medicinal plants include polyphenols, alkaloids, flavonoids, saponins, quinones, terpenes, proanthocyanins, tannins, polysaccharides, steroids, and coumarins. Among the plant phytochemicals, alkaloids represent the largest antiviral category with a broad spectrum of antiviral properties [3,27,28]. Based on our preliminary independent survey, the alkaloids comprised the largest percentage (22%) of investigated antiviral phytochemicals that were investigated against 15 families of viruses (Data not shown).

## 4. Structure and Characteristics of Alkaloids

Alkaloids are naturally occurring compounds containing carbon, hydrogen, nitrogen, and oxygen and are present in plant tissues as organic acid salts (e.g., acetic, malic, lactic, citric, or oxalic) and/or weak bases such as nicotine. In addition, some alkaloids may exist as glycosides, such as the solanum (solanine), amides (piperine), and esters (atropine, cocaine) [29]. Alkaloids can also be categorized according to their biosynthetic pathway [30] or their occurrence as heterocyclic or nonheterocyclic alkaloids [31]. They are often classified according to their molecular skeleton, for example., quinoline, indole, isoquinoline, tropane, steroidal, and pyridine and pyrrolizidine alkaloids [32].

Plant-based alkaloids are present in the form of crystalline, amorphous, non-odorous, and non-volatile compounds [31]. They are a very diverse class of secondary metabolites, encompassing an extremely divergent chemical structure. However, the structures of each of the compounds have different functional groups that give the compound its unique characteristics. The skeletal, structural, and functional diversity makes them a versatile group of secondary metabolites, meaning that they are a more significant pharmacophoric unit with desired biological activities [29,33]. According to Lipinski’s criteria, for a molecule to be drug-like, the physicochemical properties of alkaloids (molecular weight, number of NH and OH groups, the average number of N and O atoms per alkaloid, and log P) must fall within the range of the standard criteria for drug-like molecules [33,34]. Alkaloids have a low-to-moderate molecular weight (250–600 Daltons); they are water-soluble and lipid-soluble under acidic/neutral and basic conditions, respectively. Their solubility gives them a unique characteristic that is appropriate for a wide range of medical uses, as they can be transported in the protonated form and penetrate cell membranes in the neutral form. The pKa values of alkaloids range from 6 to 12 [32,35].

## 5. Targets for Antiviral Alkaloids

More recently, significant progress has been made in the screening and use of small molecules (with low molecular weight) as inhibitors of biochemical processes. Natural products, especially alkaloids, are small molecules potentially useful for this method of investigation [36], as they have been proven to interact with a wide range of biological targets. Their mechanism of action varies with their class and chemical structure [37]. Both isolated compounds and plants (or extracts thereof) containing alkaloids have been widely investigated and used. Alkaloids have been proven to interact with a wide range of biological targets [37]. Antioxidant properties, scavenging capabilities, DNA and RNA synthesis inhibition, and viral replication blockage are the main antiviral activities of many adopted phytochemical compounds, and these activities may be due to the synergistic effects of more than one mechanism [38]. It has been extensively stated that there is a significant association between the concentration and toxicity of any substance used as a drug, and lower doses of drugs are always recommended. In our preliminary literature review, we found that alkaloids have dose-dependent effects, and most of the studies carried out at concentrations lower than 10 µM showed effective antiviral activities with no significant toxicity. Thus, reviewing the acceptable practices for studying antiviral activities of alkaloids, we focused on the antiviral effects of alkaloids at concentrations in the order of magnitude of 10^−3^–10 µM, which are expected to be pharmacologically more suitable. Furthermore, we subdivided the reviewed alkaloids into two classes (viral attachment and cell entry target and viral replication target), based on the mechanisms of action. Experimental evidence of the interaction and interference of alkaloids with cellular components is summarized in Table 1.

### 5.1. Viral Attachment and Cell Entry Target

Viral cell entry is the first essential step in the viral life cycle and infection establishment since viral infection requires the transfer of the viral genome into the host cell cytoplasm, either at the plasma membrane or the endomembrane system. Virus entry and post-entry stages have been the subject of several advanced recent studies. The prevention of viral infections through the host cell is widely considered to be one of the appealing targets of effective drug development. Moreover, host cell targeting could reduce drug resistance as the target compounds would act on the host proteins [72,73]. For both enveloped and non-enveloped viruses, entry into the cell begins with an attachment to cell-surface receptors and end with the delivery of the viral genome to the cell cytoplasm or nucleus, where its replication process can start. This can occur either by fusion (for enveloped viruses) or penetration (for non-enveloped viruses) [74,75].

The entry of enveloped viruses typically involves fusion between the viral lipid envelope and the host membrane. Growing evidence suggests that alterations in the lipid membrane can block viral release and entry. The alterations can be caused by fusion inhibitors, a class of antiviral drugs that act extracellularly to prevent direct attachment and entry of viruses. They include certain lipid active compounds, a potential antiviral agent [76]. For example, LJ001 is a membrane-binding molecule that was proven to effectively inhibit the viral entry of numerous enveloped viruses. Through its nonpolar end, LJ001 penetrates viral lipid membranes and inhibits viral entry, possibly by affecting the rigidity and curvature of the membranes, thus preventing efficient virus–cell membrane fusion [77].

Virus entry is specific for host cell susceptibility and depends on viral surface proteins and receptors. The enveloped viruses use a two-step procedure to release their genetic material into the cell, typically they first bind to specific surface receptors of the target cell membrane and then fuse between the viral lipid envelope and the host membrane. Recent studies revealed that alterations in the lipid membrane can block viral release and entry, suggesting their potential as antiviral drugs. Like some other small-molecule inhibitors, the alkaloid michellamine B has previously been shown to suppress HIV infection at an early stage by inhibiting cellular fusion and syncytium formation [63]. Further, several related compounds have been identified as acting by blocking the attachment and entry of some novel viruses. For example, emetine was identified as an entry inhibitor of the Zika virus (ZIKAV) and Ebola (EBOV) viruses, disrupting lysosomal function and blocking the ZIKAV NS5 polymerase activity [48]. In ZIKAV, the fusion loop epitope, a segment of the envelope (E) glycoprotein, represents a potential therapeutic target impacting host attachment [78]. In filoviruses such as EBOV, the spike glycoprotein (S) promotes membrane fusion and plays an essential role in virus entry and contact with the cell [79].

HIV entry also involves fusion between the viral lipid envelope and the host cell. Thus, fusion inhibitors can prevent direct attachment and entry of HIV. Mcmahon et al. reported the inhibition of the HIV life cycle by michellamine B at an early stage by inhibiting RT and at later stages by inhibiting cellular fusion and syncytium formation. The same authors also revealed that michellamine B inhibited the production of RT, p24, and infectious virions in HIV-infected CEM-SS cells and freshly isolated peripheral blood cells. The inhibition of cell fusion by michellamine B may result from reversible interactions with membrane components that play a role in cell–cell fusion and focus on the entry process due to the amphoteric nature of alkaloids [63]. Besides, in another study, infection by the RT M184V HIV-1 mutant (in reverse transcriptase, RT) was successfully blocked by emetine, thereby showing a direct inhibitory effect on viral entry. It appears that the underlying mechanism of this inhibition may involve interference with intact HIV particles and block the reverse transcription reaction, which often prevents the entry of a virus to the host [48]. Emetine was also identified as an entry inhibitor of the Zika (ZIKAV) and Ebola virus diseases (EVD) by disrupting lysosomal function and blocking the ZIKAV NS5 polymerase activity [48]. Further, several related compounds have been identified as effective by blocking the attachment and entry of some novel viruses. In a recent study, a full agreement of molecular docking simulation with the experimental results was observed for the binding of berberine to the E2 glycoprotein of HCV and specific blocking of the viral attachment and entry [80]. In the case of SARS-CoV-2, spike (S) protein S2 subunit plays a key role in mediating virus fusion with and entry into the host cell. The heptad repeat 1 (HR1) and heptad repeat 2 (HR2) of the S protein can interact to form a six-helical bundle (6-HB), thereby bringing viral and cellular membranes in close proximity for fusion [81]. Recent computational studies showed that piperine and berberine alkaloids have a significant binding affinity towards the spike glycoprotein of SARS-CoV-2 and ACE2 receptor and may be useful as a therapeutic agent for restricting viral attachment to the host cells [82]. Another molecular docking and dynamic simulation study predicted a potential inhibitory effect of alkaloids on SARS-CoV-2 infection by binding to the receptor-binding domain of the SARS-CoV-2 S protein and putatively preventing it from binding to the host cell. Therefore, based on the simulation investigations, it was suggested that the alkaloids (cryptospirolepine, 10-hydroxyusambarensine, and cryptoquindoline) might have the potential to prevent SARS-CoV-2 infection [83,84]. However, their applicability as antiviral compounds against SARS-CoV-2 needs to be studied experimentally to provide strong evidence of the interaction and inhibition.

**Figure 1:**
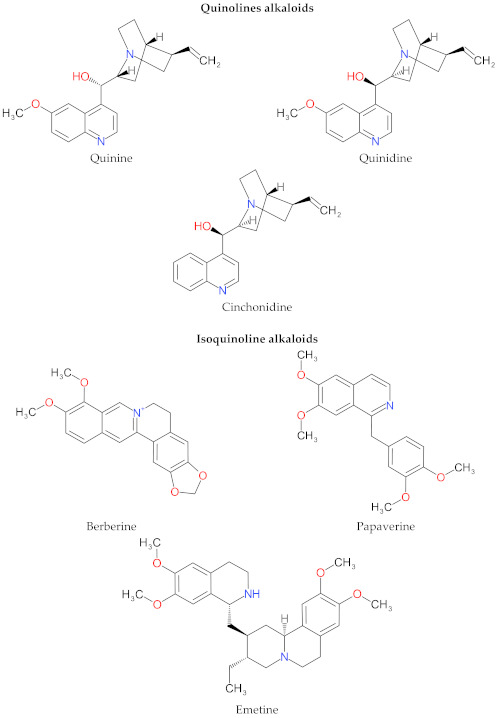

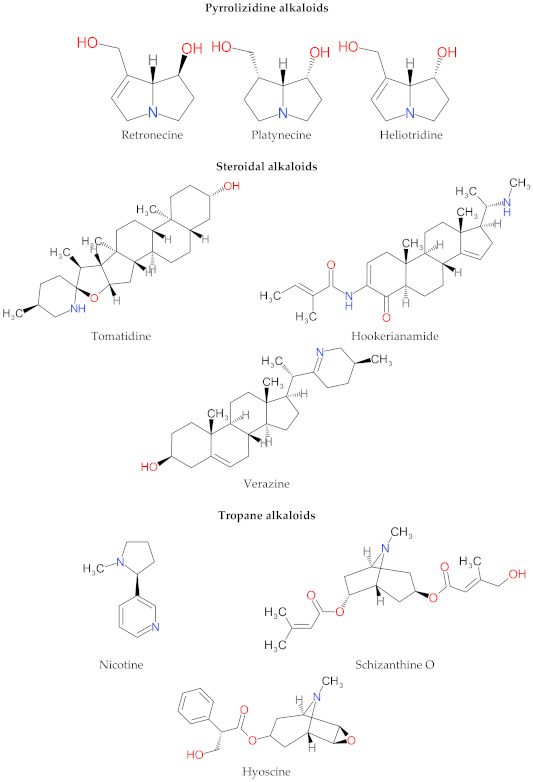

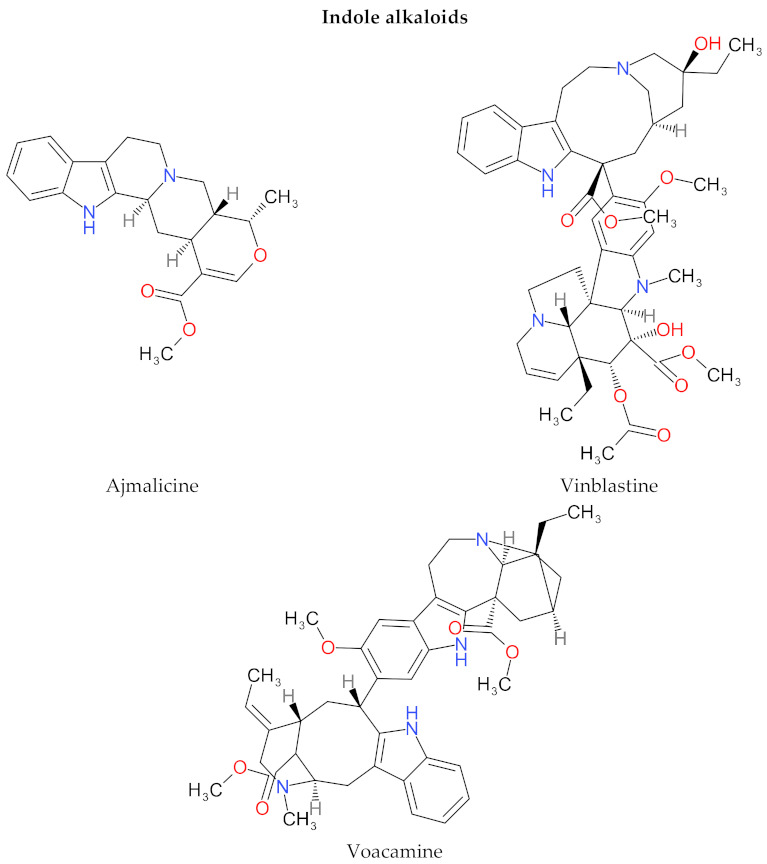
Chemical structure of alkaloids.

Several studies suggested that many natural alkaloids effectively interfere with the cell membrane (even though this membrane is not the final target) and indirectly function by binding to the viral glycoprotein. The amphiphilic nature of alkaloids is likely to provide a rationale for their antiviral activities, particularly as an entry inhibitor preventing viral attachment. Chemically, amphiphilic alkaloids have a lipophilic molecular framework and a specific functional group (Figure 1). Amphiphilic alkaloids have a predominantly hydrophobic character that enables permeation of the cell membrane and then interaction with the membrane components such as proteins or lipids at the periphery of the cells. They can also interact with various receptors and ion channels, resulting in physicochemical property changes of the lipid bilayer and in the activities of different receptors, ion channels, and enzymes [85,86,87,88,89]. Research on alkaloid–cell membrane interaction explained the possible mechanism of alkaloids penetration through the cell membrane. The results suggested that the alkaloid–lipid interaction affects the lipid headgroup structure by weakening the lipid headgroup–headgroup H bonds and enhancing the electrostatic attraction between the alkaloids and the lipid phosphate groups, facilitating the alkaloid passage between the headgroups [85].

However, the cell membrane is the main target site of specific fusion alkaloid inhibitors leading to the disruption of the glycoprotein within the virion envelope, surface receptors, and the disruption of the attachment of viruses to host cells, reducing the possibility of effective virus–host interaction [90,91,92,93,94]. Therefore, conformational rearrangements of the viral membrane lipids strongly influence viral membrane fusion and may even be exploited for the design of broad-spectrum antivirals against a wide variety of enveloped viruses.

### 5.2. Viral Replication Target

As the nucleic acids are the most desirable target for inhibiting the specific viral replication step, therefore, research on their structural and conformational changes resulting from their interactions with small molecules is currently a matter of intense interest [95]. Many alkaloids attack the genetic material and enzymes involved in the viral life cycle through multiple mechanisms and interrupt their natural biological functions. In this section, we review the antiviral activities of natural alkaloids at the molecular level, specifically targeting the synthesis of DNA and RNA, as well as proteins.

#### 5.2.1. Inhibition of DNA and RNA Synthesis

Viruses vary in form and complexity. They consist of genetic material, DNA or RNA, either of which may be single or double-stranded, surrounded by a coat composed of protein. Viruses encode several functions necessary for their life cycle. However, they are entirely dependent on the protein synthesis machinery of their host cells [96]. First, viruses use cellular ribosomes to translate their viral mRNA into structural proteins, envelope proteins, and viral enzymes [97]. RNA viruses replicate in the cytoplasm and rely on their RNA genome as a template for the synthesis of additional RNA. Three types of RNA must be synthesized during the replication process, including the genome, a copy of the genome, and mRNAs [98]. Viral mRNA transcripts of DNA viruses are produced within the cell nucleus through the host’s DNA-dependent RNA polymerase II. Replication of a DNA virus is highly accurate as the DNA polymerase checks the copied sequences (proofreading) and removes most mismatches [97].

The replication cycle of viruses is complex and generally involves three main steps: initiation of the infection, genome replication and expression, and finally, the release of mature virions from the infected cell [99]. Replication cycles during the viral life cycle represent apparent targets for intervention. Cumulative evidence suggests that HIV reverse transcriptase is the major anti-HIV target involved in viral replication [100]. Chain termination inhibiting the herpes virus replication process [101] is another example, as well as polyamine depletion, which restricts replication, translation, and packaging of diverse RNA and DNA viruses [102,103]. Viral proteases are very successful, and their inhibitors are actively developed [104]. Current evidence suggests that RNA-dependent RNA polymerase could be a target for the inhibition of SARS CoV-2 [105].

Inhibiting nucleic acid synthesis sounds like a great strategy for antiviral activity. Nucleic acid intercalation is a well-known process for interrupting DNA-associated pathways as they perturb DNA structure, which can, in turn, influence DNA processing. This alteration in structural and mechanical properties of DNA could offer broad-spectrum antiviral agents that are more effective than target-specific compounds in preventing multiple emerging infectious diseases. Generally, DNA–ligand H noncovalent interactions require the presence and insertion of an appropriate size and chemical nature of moieties between adjacent DNA base pairs [106]. These ligands are mostly aromatic, planar, and polycyclic and, therefore, often make good nucleic acid stains/dyes [107]. DNA and RNA intercalation is of great interest in the context of many alkaloids and represents a good inhibition activity. A series of studies combining biochemical methods with numerous interdisciplinary techniques provided clear evidence on alkaloid–nucleic acid interaction effects. It has been previously established in trypanosomes that some of the alkaloids can effectively intercalate with DNA or DNA processing enzymes such as DNA polymerase I and reverse transcriptase. Other alkaloids have profound effects on the RNA polymerases and DNA topoisomerase, thus impairing base-pairing and blocking DNA replication, DNA repair, and transcription, which remain a vital drug target [108]. Many of these intercalators have planar geometry with aromatic moieties that can smoothly fit between AT and GC base pairs [109]. Intensively studied DNA-alkaloids intercalators include berberine, emetine, sanguinarine, β-carboline, quinine, skimmianine, cinchonine, and dictamine [29]. The intercalation of berberine and palmatine alkaloid molecules within a triple-helical RNA structure, poly(U), poly(A), poly(U), was experimentally demonstrated using various biophysical and calorimetric techniques. This study brought an advanced understanding of the specific binding interaction of alkaloids to RNA structures, particularly triplexes, and may be useful in developing RNA-targeted therapeutics [110]. Although DNA–alkaloid intercalation has been extensively studied and successfully demonstrated as a broad biological activity in different organisms, only a few studies have been carried out on the antiviral effects. The reviewed studies suggested that intercalation of an alkaloid, such as buchapine, colchicine, acronycine, and lycorine, into DNA, could serve as a promising inhibitory mechanism against HIV, HSV, and human cytomegalovirus (HCMV) replication, via structural changes in the DNA that could lead to irreparable damage in the genetic material of the virus [47,111,112].

The capacity of several alkaloids to inhibit DNA and RNA synthesis in multiple virus families via different mechanisms has been experimentally proven (Table 1). For example, oliverine interferes with and inhibits HSV-1 at the early stage of replication via the suppression of DNA synthesis [63]. The lycorine inhibits the viral RNA-dependent polymerase (RdRp) of ZIKAV and dengue virus (DENV) [60]. Emetine exhibits remarkable inhibitory activity by blocking the HIV reverse transcriptase and reaching an 80% reduction in HIV-1 infection, with a low cytotoxic effect [49]. Emetine also inhibited viral polymerases [86] and the RNA synthesis of DNA and RNA viruses such as DENV, buffalopoxvirus (BPXV), bovine herpesvirus 1 (BHV-1), and Newcastle disease virus (NDV) without generating drug-resistant virus variants. This suggests that emetine could provide significant therapeutic value against some of these viruses without inducing an antiviral drug-resistant phenotype [47,113]. Tomatidine is a steroidal alkaloid derived from the stem and leaves of unripe, green tomatoes. It has been recently described as having antiviral activity towards CHIKV infection. This is commonly achieved by interfering with the production of infectious viral particles and inhibiting replication after virus attachment and cell entry [69]. Camptothecin is a DNA topoisomerase 1 (TOP1) inhibitor that inhibits enterovirus 71 (EV71) RNA replication and translation, altering the template DNA and thus affecting the synthesis of DNA and RNA. Alternatively, camptothecin may inhibit the activity of an enzyme involved in DNA repair, such as DNA ligase degrading the DNA, thereby representing irreparable DNA damage [44].

#### 5.2.2. Inhibitors of Protein Synthesis

Targeting the virus life cycle at the protein synthesis level with alkaloids demonstrates the molecular basis of specificity in nucleic acid–alkaloid interactions, for example, the associations of alkaloids with different nucleic acids, such as ribosomal ribonucleic acid (rRNA), transfer ribonucleic acid RNA(tRNA), or messenger RNA (mRNA) are different and specific. Some alkaloids were found to be specifically toxic to plasmodia, as they prevented the biosynthesis of plasmodial DNA by preventing the integration of building blocks into plasmodial DNA [114,115]. A typical example of this group of alkaloids is omacetaxine. Omacetaxine is a natural homoharringtonine alkaloid from the Cephalotaxus genus that has been approved by the FDA as a protein synthesis inhibitor drug [116].

Several alkaloids have been proven to be potent inhibitors of protein synthesis in various families of viruses. A likely mode of action is blocking the ribosomal protein. The 40S ribosomal subunit appears to be the site of a specific receptor for emetine, which inhibits viral translation [117,118]. Other studies have reported the multiple actions of alkaloids, for example, emetine, which can block the reverse transcriptase of HIV, and inhibit viral polymerases, in addition to suppressing the viral translation by disrupting the 40S ribosomal protein S14 and blocking the synthesis of the viral proteins in infected cells [47,51,109,113]. Fresno et al. reported that some alkaloids can block the formation of peptide bonds and inhibit the enzyme binding of aminoacyl-tRNA [119].

As summarized in Table 1, the effect of alkaloids on specific steps of the viral life cycle has been widely reported. For example, a possible inhibitory mechanism of emetine on viral genome replication could be due to direct inhibition of viral protein synthesis. Since virus replication requires the presence of viral structural and non-structural proteins, their inhibition may eventually block viral genome replication [49]. Efficacy and mechanism of low dose emetine as a human cytomegalovirus inhibitor in a mouse model (HCMV) was also reported. The inhibition of HCMV by emetine occurred after virus entry but before DNA replication and resulted in a decreased expression of viral proteins, which are essential for the production of infectious HCMV. Synergistic virus inhibition was achieved when emetine was combined with ganciclovir [51]. Emetine has also been shown to block the synthesis of the viral proteins in HIV-1, suggesting the importance of further studies to evaluate the potential use of this compound as an anti-HIV inhibitor. Earlier studies demonstrated the inhibition of encephalomyocarditis virus protein synthesis by emetine and harringtonine alkaloids. The observed inhibition of protein synthesis occurred during the elongation step [120]. Similarly, polypeptide chain elongation was blocked by the interaction of lycorine with the C-terminal region in the polyproteins during the elongation step, and lycorine thus inhibited protein synthesis of EV71 [121]. Lycorine inhibits protein synthesis in eukaryotic cells by inhibiting the peptide bond formation step [122]. Lycorine inhibits the transpeptidation reaction by suppressing the coupling of the N-acetyl leucyl residue from UACCA-acetyl leucyl to puromycin [123]. Berberine inhibits protein synthesis through viral protein trafficking or maturation and TNF-α and PGE2 [42]. Berberine also interferes with the viral immediate-early 2 (IE2) protein of cytomegalovirus and remarkably inhibits the synthesis of the proteins essential for regulating the expression of many host genes and the production of infectious HCMV [40]. Quinine significantly suppresses HSV-1 protein synthesis, inhibits early steps of the influenza A virus (H1N1) duplication cycle [124], inhibits DENV by decreasing viral RNA and protein synthesis, and amends the expression of the genes connected to inborn immune response [125]. Alkaloid antiviral effects dramatically prevent the completion of the viral growth of different strains in infected cells. Most of these phytochemicals attack cellular enzymes and viral proteins. The findings suggest that alkaloids target the cellular and viral components with multiple action mechanisms, acting as specific or broad-spectrum inhibitors of viral replication. Every essential step in the virus life cycle is an attractive target for antiviral alkaloids. Remarkably, the antiviral activities of alkaloids inhibit replication by targeting cellular enzymes and protein synthesis. Some alkaloids have dual cellular mechanisms, such as fusion and replication inhibition. In addition, some alkaloids show specific inhibition of protein synthesis, mostly at the peptide bond formation step. Most studies confirmed protein synthesis inhibition through action on the RdRp 40S ribosomal subunit and some other specific virus receptors. Ultimately, the interference of alkaloids with nucleic acids resulted in an alteration in the nucleic acid sequence, leading to blockage of the transfer of messages important for protein synthesis, thereby preventing the protein production required for many viral infections.

## 6. Toxicology and Side Effects

Irrespective of their established antiviral activities, some alkaloids are recognized as too toxic and are banned for animal and human use. The harmfulness of alkaloids, as with other drugs, depends on the dose. However, it is difficult to make conclusions about the overall significance and utilization of alkaloid compounds considering the lack of specific data on the toxicokinetics of the examined alkaloid compounds in clinical studies. Nevertheless, the compounds highlighted in this review displayed no harmful effects in in-vitro studies at lower concentrations. Thus, in the next section, attention will be paid to the major restrictions of the studied alkaloids in terms of their toxicity in preclinical and clinical trials.

As described above, camptothecin (CPT) has a confirmed and efficient antiviral activity. The main disadvantage of using CPT as an antiviral agent is its cytotoxicity, found in HT-29 cells with an IC50 value of 10 nM. CPT induces DNA impairment at concentrations as low as 51 nM in whole cells and 12 nM in isolated nuclei in in-vitro studies. Furthermore, results from clinical trials in the treatment of progressive gastrointestinal cancer were not as promising and were discontinued in the 1970s [126]. Attention will certainly remain focused on camptothecin due to both the present pharmaceutical possibilities and the additional analogs or derivatives of camptothecin. New progress in CPT-related antiviral molecules represents the synthesis of camptothecin associated analogs with notably low cytotoxicity [55,127]. A new camptothecin-20-O-propionate hydrate (CZ48) was created and presented as an anticancer agent against 19 human tumor xenografts, lymphoma, and solid tumors [128]. No toxicity was experienced in the in-vitro study. As a result of the harmless nature of the drug in rats, the highest tolerated dose was not reached [119]. Continuing clinical safety-related trials were carried out in a single-arm, non-randomized viability and phase I trial of 20 camptothecin propionate administered run orally to 65 participants at the Cancer Therapy and Research Center at the UT Health Science Center at San Antonio, Texas [129]. A clearer picture of these side effects and the toxicity will be known once the results of these clinical trials are released. A recent in vitro investigation of nanoencapsulated camptothecin prevented HCMV replication at a low concentration suggesting that the delivery method could alter the toxicity [130]. The extensiveness of preclinical and clinical data on CPT analogs will be needed to help direct the preparation and scientific development of a lead therapeutic compound.

Michellamine B is the major in vitro active anti-HIV component. An earlier update on plant-derived components used for the treatment of HIV based on preclinical assays showed that the effective antiviral activities of michellamine B could only be attained at close to neurotoxic dose levels. For example, an effective dose of 25 mg per kg has been regarded as too toxic for clinical trials, and therefore the additional studies for the treatment of HIV infections in the United States were suspended [131]. Notwithstanding the cessation of the development of michellamine B as a possible anti-HIV agent, there is still the potential to develop less toxic and simpler analogs of michellamine B, which are currently being widely examined [132,133].

One of the observed examples of alkaloid intercalating into DNA is colchicine which was approved by the FDA in 1961 and is generally used in the management of gout [134]. Colchicine may exert an immuno-suppressive effect. However, the tolerability and safety of colchicine have been confirmed in huge randomized cardiovascular trials [135]. There are now 28 continuing clinical trials of colchicine to examine its positive effects on moderate to severe COVID-19 infections [136]. A phase 3, randomized, double-blind, placebo-controlled multicenter study to assess the safety and efficacy of colchicine in 4065 adult patients diagnosed with COVID-19 infection is continuing, and as the outcome of these trials become accessible, it should become clearer whether there is a benefit to using colchicine in patients with COVID-19. Another recently completed study comprising 36 COVID-19 patients showed that colchicine reduced the length of both additional oxygen therapy and hospitalization, and the drug was safe and well-tolerated [137].

Emetine is an alkaloid of substantial medicinal value. Despite its antiviral strength, its curative use has several side effects, and the use of the drug has been diminished by dose-dependent toxicity. Cardiotoxicity is the most dangerous and serious hostile effect of emetine. The clinical indications are hypotension, dysrhythmias, tachycardia, and cardiomyopathy. Electrocardiographic anomalies happen in 60–70% of cases [138,139]. A study looking at emetine dihydrochloride hydrate as a possible candidate for use against malaria was recently undertaken. In this study, a possible route to connect the nanomolar (median effective dose, ED50, of 47 nM) antimalarial efficiency of this inexpensive natural product was described to minimize the formerly reported dose-related toxicity of the drug [140].

Lycorine is the most common *Amaryllidaceae* alkaloid and has an extensive array of bioactivities. In disparity with its possible health-promoting effects, it is assumed to be the cause of plant poisoning in animals and humans and exhibits numerous side effects. Results proved that lycorine at 1.0 mg/kg body weight causes emesis and nausea in animals and humans due to poisoning [141]. Lycorine as a potential natural candidate for an anticancer drug, was efficient in a very low, single-digit micromolar concentration; the IC50 value generally did not exceed 7.5 μM and it was highly tolerable with negligible toxicity [142,143].

Omacetaxine, a protein synthesis inhibitor, is used for treating leukemia. In a second phase of clinical trials, omacetaxine was tested on 103 patients with chronic myeloid leukemia (CML) with the BCR-ABL T315I mutation, and results showed that omacetaxine might offer an effective and safe treatment for these patients. Omacetaxine has been found to improve persistence in patients with persistent CML and resistance to many tyrosine kinase inhibitors and was therefore accepted for usage in the United States in 2012 [144,145].

## 7. Conclusions

Much research has been dedicated to combating the pandemic threat of viruses, which can have devastating effects on humans, plants, and animals. Viruses have initiated the deadliest and most horrifying diseases the world has seen. The most recent example of this is the ongoing coronavirus disease 2019 (COVID-19) pandemic. With the rapid development of new resistant viral strains, there is a need to identify antivirals that target these strains with more effectiveness and less toxicity.

Natural plant research has previously resulted in the development of many effective drugs. Some alkaloids are already used in well-known medicines with known action mechanisms and expected health effects. There are many approved alkaloid-derived drugs for different diseases (including infectious ones), such as the antimalarial drug hydroxychloroquine. However, the principal reason that some alkaloids are presumed to be promising antiviral candidates could be their molecular basis of specificity and their ability to target multiple virus families with different mechanisms of action. Therefore, a great deal of work has been carried out to explain the mechanisms of action and the effective and lethal doses of alkaloids to investigate and expand their use as antiviral drugs. These brief, reliable, and replicable scientific facts, along with the current availability of state-of-the-art analytical technology, should be explored translated into actionable clinical trials of natural products.

## Figures and Tables

**Table 1:** Antiviral activities of alkaloids.

Compound	Source	Virus	Dose IC_50_ or EC_50_	Activity	Experiment	Ref
Anisomycin	Pure	DENV, ZIKAV	7.6 nM 15.9 nM	Inhibits replication	In vitro/In vivo	[39]
Berberine	*Berberis vulgaris (Berberidaceae)*	HCMV	0.68 μM	Inhibits the virus replication cycle between viral attachment/entry and genomic DNA replication	In vitro	[40]
		HSV-1 and 2	6.77 and 5.04 μM			[41]
	*Hydrastis Canadensis*	H1N1	0.01 μM	Inhibited production of proteins		[42]
Buchapine,	*Euodia roxburghiana*	HIV-1	0.94 μM	Inhibit reverse transcriptase	In vitro	[43]
	*Ophiorrhiza mungos*	VACV	10 μM,	Inhibition of viral DNA synthesis	In vitro	[44]
Camptothecin	*Camptotheca acuminata*	EV71	10 μM	Inhibits viral RNA replication and translation	In vitro,	[45]
Colchicine	*Haplophyllum tuberculatum, Colchicum autumnale*	HIV 1	1–10	Inhibit replication by DNA intercalation	In vitro	[46]
Emetine	*Cephaelis ipecacuanha A.*	RNA, DNA viruses	3.03 ng	Inhibition of viral protein synthesis	In vitro/In vivo	[47]
		ZIKAV, EVD	10 μM	Inhibition of ZIKAV NS5 polymerase activity and disruption of lysosomal function, inhibits EBOV entry andreplication of DNA viruses	In vitro/In vivo	[48]
		HIV	0.72 μM	Inhibits HIV-1 Replication by interfering with reverse transcriptase activity	In vitro	[49]
		SARS, MERS	0.0135 μM	Replication inhibition	In vitro	[50]
		HCMV	0.0087–0.04 μM	Inhibition on ribosomal processing S14 (RPS14) binding	In vitro	[51]
		SARS-CoV-2	0.46 μM	Replication inhibition	In vitro	[52]
Fangchinolin	*Stephania tetrandra*	HCV	10 µM	Suppressed the replication and inhibited viral S and N protein expression	In vitro	[53]
Gliotoxin	*Pure*	HI	10 μM	Inhibits intracellular replication	In vitro	[54]
Hemanthamene		H5N1	4·15 μM	Inhibits the translocation of the ribonucleoprotein complex	In vitro	[55]
Homorringtonine	*Cephalotoxus fortune*	SARS-CoV-2	2.55 μM	Replication inhibition	In vitro	[52]
Isatindigobisindoloside F.	*Isatis indigotica*	CVB3	8.4 μM	Inhibit the replication	In vitro	[56]
Lycorine	*Clivia miniate*	ZIKAV	0.01 to 10 μM	Inhibits viral replication by restraining RdRp activity	In vitro/In vivo	[57]
		HCV	6.10 μM	Inhibits viral replication by the expression of hsc70 in host cells	In vitro	[58]
		H5N1	0·52 μM	Inhibits the translocation of the ribonucleoprotein complex	In vitro	[59]
		DENV	0.4 μM	Suppression of viral RNA replication	In vitro	[60]
	*L.radiata*	SARS-CoV	15.7 nM	Viral inhibition	In vitro	[61]
Manzamine	*Acanthostrongylophora*	HSV-1	1 µM	Repressed ICP0 transcription	In vitro	[62]
Michellamines B	*A. korupensis*	HIV	1 μM	Inhibiting cellular fusion and syncytium formation	In vitro	[63]
Michellamines A, B, and C	*Ancistrocladus korupensis*	HIV1, HIV2	2–10 μM	Complete inhibition of the replication and cytopathic effects of HIV	In vitro/In vivo	[64]
Oliverine	*Polyathia oliveri;*	HSV-1	7.5 μM	Inhibition of viral DNA synthesis	In vitro	[65]
Polycitone A	*Polycitor sp*	HIV, MLV, MMTV	295 nM	Potent inhibitory activity on both RNA- and DNA-directed DNA polymerases	In vitro	[66]
Reserpine	*Pure*	SARS	3.4 μM	Inhibits 3CL protease and viral entry	In vitro	[67]
Schumannificine	*Schumanniophyton magnificum;*	HSV	1.6 μM	Irreversible binding to gp120	In vitro	[53]
Tetrandrine,	*Stephania tetrandra*	HCV	10 µM	Suppressed the replication and inhibited viral S and N protein expression	In vitro	[53]
Thalimonine	*Thalictrum simplex*	H7N7	0.1 µM	Inhibits viral reproduction	In vitro	[68]
Tomatidine	*Skin and leaves of tomatoes*	CHIKV	1.3 µM	Inhibits infection of three different CHIKV genotypes	In vitro	[69]
		DENV	0.82 μM	Inhibit replication	In vitro	[70]
18-methoxycoronaidine	*Tabernanthe iboga*	HIV-1	9.5 μM	Inhibit the replication of primary isolates of HIV	In vitro	[71]

## Data Availability

Not applicable.

## Abbreviations

CHIKV	Chikungunya virus
CVB3	Coxsackievirus B3
DENV	Dengue virus
EV71	Enterovirus 71
EVD	Ebola virus disease
H1N1	Influenza A virus
H5N1	Asian avian influenza A virus
H7N7	Avian influenza A virus
HCMV	Human cytomegalovirus
HCV	Hepatitis C virus
HIV	Human immunodeficiency virus
HSV	Herpes simplex virus
MERS	*Middle East respiratory syndrome*
MLV	Murine leukemia viruses
MMTV	Mouse mammary tumor viruses
SARS	*Severe acute respiratory syndrome*
SARS-CoV-2	Severe acute respiratory syndrome coronavirus 2
VACV	Vaccinia virus
ZIKAV	Zika virus
